# Early changes in physiological variables after stroke

**DOI:** 10.4103/0972-2327.44555

**Published:** 2008

**Authors:** Andrew A. Wong, Stephen J. Read

**Affiliations:** * Department of Neurology, Princess Alexandra Hospital, Brisbane, Queensland, Australia; # Department of Neurology, Royal Brisbane and Women's Hospital, Brisbane, Queensland, Australia; ^ Central Clinical School, School of Medicine, University of Queensland, Brisbane, Queensland, Australia

**Keywords:** Blood glucose, blood pressure, body temperature, cerebrovascular disorders, oxygen

## Abstract

Several aspects of physiology, notably blood pressure, body temperature, blood glucose, and blood oxygen saturation, may be altered after an ischemic stroke and intracerebral hemorrhage. Generally, blood pressure and temperature rise acutely after a stroke, before returning to normal. Blood glucose and oxygen levels may be abnormal in individuals, but they do not follow a set pattern. Several aspects of these physiological alterations remain unclear, including their principal determinants - whether they genuinely affect prognosis (as opposed to merely representing underlying processes such as inflammation or a stress response), whether these effects are adaptive or maladaptive, whether the effects are specific to certain subgroups (e.g. lacunar stroke) and whether modifying physiology also modifies its prognostic effect. Hypertension and hyperglycemia may be helpful or harmful, depending on the perfusion status after an ischemic stroke; the therapeutic response to their lowering may be correspondingly variable. Hypothermia may provide benefits, in addition to preventing harm through protection from hyperthermia. Hypoxia is harmful, but normobaric hyperoxia is unhelpful or even harmful in normoxic patients. Hyperbaric hyperoxia, however, may be beneficial, though this remains unproven. The above-mentioned uncertainties necessitate generally conservative measures for physiology management, although there are notably specific recommendations for thrombolysis-eligible patients. Stroke unit care is associated with better outcome, possibly through better management of poststroke physiology. Stroke units can also facilitate research to clarify the relationship between physiology and prognosis, and to subsequently clarify management guidelines.

## Introduction

It is well established that stroke unit care improves outcome.[1] How this is achieved remains unclear, but close monitoring and maintenance of physiological homeostasis may contribute significantly to this benefit.[2–4] However, our understanding of the changes in the main modifiable physiological parameters, namely blood pressure, body temperature, blood glucose and blood oxygen saturation, and the impact that these changes have on stroke outcome remains incomplete. Threshold levels for instituting treatment to modify these parameters, targets to be achieved with treatment and the effectiveness of such treatment remain uncertain. In this paper, we will review what is known about acute poststroke changes in these physiological variables, the causes of these changes, their prognostic significance, and the effects of treatment to modify these parameters.

## Blood Pressure

### Ischemic stroke

Changes in blood pressure (BP) after an ischemic stroke are important because there is impairment of cerebral autoregulation,[5] which, in normal circumstances, acts to maintain constant cerebral blood flow despite changes in systemic BP. With impairment of autoregulation, changes in systemic BP may affect cerebral perfusion, especially in the penumbral tissues, and may therefore affect the survival of ischemic tissue and the neurological outcome.

Both the systolic and diastolic BP are higher after stroke. It appears to rise acutely at the time of stroke.[6] Blood pressure then falls over the next 7-10 days,[7,8] with most of this fall occurring within the first 1-2 days.[9,10]

The mechanisms driving these BP changes are uncertain, though there are several plausible explanations. A relationship between severe stroke and high poststroke BP has been documented,[11] although others have found that severe stroke is associated with a lower BP than stroke of mild to moderate severity.[10,12] How stroke severity might modulate BP is unknown. Acute psychological stress related to the process of hospital admission has been suggested to be a cause,[13] although a study in which high BP was documented after admission with stroke but not after medical admissions with other acute problems suggests that the BP elevation is stroke-specific.[14] However, since psychological stress cannot be quantified, it is possible that the stroke group experienced more psychological stress than the control group. A neuroendocrine stress response may contribute and BP has been correlated with salivary[15] and serum[16] cortisol and urinary catecholamine[16] levels, although others have found no correlation between serum cortisol and BP.[17] Inflammation may also play a role, one group finding an association between BP and C-reactive protein (CRP) levels.[18] Inflammation and a neuroendocrine stress response may be linked, given the reported relationship between interleukin 6 (IL-6) and cortisol levels.[19] Other associations with high poststroke BP include a history of hypertension[10] and the requirement for antihypertensive medications,[10] although the latter is likely to be an effect rather than a cause of higher BP.

It is hypothesized that BP elevations after ischemic stroke represent an adaptive response that helps to maintain the cerebral blood flow and perfusion of the ischemic penumbra, despite loss of cerebral autoregulation.[20] Conversely, it is of clinical concern that excessive rise in BP could lead to neurological deterioration from hemorrhagic transformation, especially in the presence of a damaged blood brain barrier.

The association between BP and the outcome of stroke is unclear, with poor outcome being associated with either absolute high[11] or low[21] BP, or having no association with BP.[22] Recent studies have identified a U-shaped relationship between BP and outcome, with poor outcome at either end of the BP spectrum.[12,23] Methodological issues, including cohort selection, the timing of BP measurements, and the timing and selection of endpoints, might contribute to the differing conclusions drawn by investigators.

The prognostic impact of BP levels appears to vary between ischemic stroke subtypes. One study found a U-shaped relationship between admission BP and mortality only in cardioembolic but not lacunar strokes[24] and another found that the relationship between admission BP and mortality in lacunar stroke was linear, with no harm resulting from low BP.[23] These findings might be explained by the lack of an ischemic penumbra in a lacunar stroke,[25] where low BP might not significantly worsen hypoperfusion and therefore, tissue survival in the ischemic territory.

Lower admission BP has also been associated with poor outcome in thrombolysis-eligible patients.[26] Patients eligible for thrombolysis must present within three hours of stroke onset, when there is likely to be a large penumbra and, therefore, a proportionately greater vulnerability to hypoperfusion. However, thrombolysis protocols also exclude patients with high BP (>185/110 mm Hg); so any deleterious effect of high BP might not be evident in these cohorts.

One study also showed that cardioembolic stroke patients had lower BP in the first 24 h and poorer outcome,[21] as compared to those with non-cardioembolic strokes, although this may merely reflect the impact that more severe stroke in cardioembolic patients has on both BP levels and outcome.

Although absolute systolic blood pressure (SBP), diastolic blood pressure (DBP) and mean arterial pressure (MAP) values are the most commonly studied indices of BP, other aspects of BP have been linked with stroke outcome. Elevated pulse pressure,[27] wide fluctuations in both SBP and DBP within the first three hours of stroke,[28] greater variability of the DBP within the first 72 hours[26] and increased beat-to-beat SBP and DBP variability within 24-72 hours of stroke[29] have all been associated with poor prognosis.

The normal circadian variation in BP, where nocturnal BP falls relative to daytime BP (dipping), may be disrupted after stroke.[30,31] This is possibly more common after right-sided[32] or insular or thromboembolic[33] infarction, and may recover in the subacute and chronic phases.[16] There is some evidence that loss of dipping may be associated with poor outcome[34] and that its preservation is associated with better outcome.[35] There are no trials investigating the effects of maintaining a nocturnal BP dip, although there is some interest in the chronotherapeutic maintenance of circadian rhythms in other situations.[36]

The prognostic significance of dynamic fluctuations in postadmission blood pressure varies. Poor outcome may be seen with spontaneous falls in systolic[37] and diastolic[38] BP; it can also be seen with spontaneous BP elevations.[11] Conversely, good outcome has been seen with spontaneous BP falls.[39] This has resulted in the investigation of both pharmacological BP elevation and reduction as potential strategies for the treatment of acute stroke.

Therapeutic BP elevation has been studied particularly in cases where relative hypoperfusion was suspected. Such cases include patients with lower BP (SBP <140 mm Hg),[40] clinical deficits that fluctuate in parallel with BP changes[41] or perfusion-diffusion mismatch (a marker for the presence of penumbral tissue) on MRI.[42] In these small studies, BP elevation was shown to be safe and to partially reverse neurological deficits.[40–42] Pharmacological BP elevation may be beneficial in carefully selected stroke subgroups, but this requires further study.[43]

Pharmacological BP lowering after ischemic stroke remains a controversial topic. Long-term BP lowering is clearly beneficial, significantly reducing the incidence of recurrent stroke events.[44] However, what remains uncertain is the optimum timing of the initiation of therapy; specifically, whether it is safe to initiate BP therapy within the time window for the persistence of penumbral tissue, with the attendant theoretical risk of affecting penumbral salvage and overall neurological outcome. Whether to continue pre-existing antihypertensive therapy is also unclear and the subject of an ongoing study.[45]

Caution is suggested by the results of research on the calcium channel blocker nimodipine, which, when studied as a neuroprotective agent, produced a worse outcome, possibly as a result of BP lowering in the treated group,[46] although this effect may have varied, depending on the stroke subtype.[47] Conversely, the ACCESS study was terminated prematurely when it was found that early treatment with candesartan was associated with a reduction in 12 month mortality and recurrent vascular events, despite there being no significant difference in the BP between treatment arms; however, no effect on early functional outcome was seen.[48] Some antihypertensive agents may lower systemic BP without affecting cerebral blood flow, as seen in the studies of perindopril[49] and glyceryl trinitrate.[50] Such agents may be safe to use early, or even beneficial; however, this remains unproven.

Some unrelated interventions and even routine activities can influence systemic BP after stroke. Insulin therapy,[51] therapeutic hypothermia[52] and experimental neuroprotective agents[46] may alter BP, potentially affecting the results of clinical trials; therefore, knowledge of the relationship between BP and prognosis is critical for the accurate interpretation of trial results. Even elevating the head of the bed to 15° or 30° can cause the MAP and cerebral perfusion pressure to fall significantly.[53] In another study, after a meal, SBP fell >10 mm Hg in a quarter of the stroke patients.[54] Clarifying whether these alterations in systemic and cerebral pressure are clinically significant is important, given that these activities are potentially modifiable in almost all stroke patients.

### Intracerebral hemorrhage

Blood pressure also rises after intracerebral hemorrhage (ICH), perhaps even more than after an ischemic stroke, and falls over the next seven days.[55] It is also possible that the rise may sometimes precede and trigger the ICH.[56]

The mechanisms behind BP changes after ICH remain uncertain. Because there is no ischemic penumbra around hematomas,[57] the BP rise is unlikely to be adaptive, especially as autoregulation seems to be preserved after ICH, and changes in systemic BP are not completely transmitted to the cerebral circulation.[58]

A rise in BP may also be harmful, higher BP having been associated with hematoma enlargement[59] and poor prognoses.[60]

Systemic BP, however, has not been universally shown to affect hematoma enlargement[61] or ICH prognosis[62] and lowering of BP has not yet been shown to improve prognosis,[63] although prospective trials are ongoing.[64]

## Temperature

### Ischemic stroke

Body temperature rises by about 0.2°C over the first 24-36 h[65,66] after an ischemic stroke. The temperature rise is greater after moderate to severe stroke (0.35°C in a cohort with National Institutes of Health Stroke Scale (NIHSS) scores ≥ 6).[66] Boysen and Christensen, for example, observed a temperature rise only in those with severe stroke (Scandinavian Stroke Score, SSS ≤ 25), with no temperature change after mild to moderate stroke.[67] They also found that the admission temperature was more often low (<36°C) after severe stroke, possibly due to immobility and low environmental temperatures.[67] Higher temperatures have also been seen in cohorts where severe stroke was defined by large infarct volumes.[68]

Following this rise, temperatures appear to fall over the first five days after stroke,[69] although the rate of normalization has not been specifically studied.

The normal circadian rhythm of temperature is also disrupted after severe stroke, possibly due to impaired consciousness or physical inactivity[70] and this may be associated with a poor prognosis,[71] although it is not clear that the relationship is independent of stroke severity.

Although the association between temperature elevation and stroke severity seems firm, the mechanism through which this effect is generated remains unclear.

Infection is common after stroke, affecting 25-35% of the patients.[72,73] In some cases, the infection may precede the stroke and has been implicated in the causation.[74] Vila *et al*. found that IL-6 levels were associated with higher temperatures and larger infarctions in a cohort of patients without infection.[75] Audebert *et al*. found that the CRP, white cell count and temperature were high in the acute phase of stroke, but normalized within days of successful thrombolysis.[76] These results suggest that ischemic or infarcted brain tissue promotes inflammation and hyperthermia. There may also be a role for a neuroendocrine stress response, with a correlation between cortisol levels and serial temperatures being documented.[17,77] Higher temperatures are more common in those requiring antipyretics, but this is more likely to have been a consequence of higher temperatures rather than a cause.[66,67]

Low admission temperatures have been correlated with poor prognosis,[67] although this relationship was not shown to be independent of stroke severity. Most investigators, however, have found the opposite effect.

In one meta-analysis, high poststroke temperatures increased mortality with an odds ratio (OR) of 1.19[78] and this relationship has been replicated subsequently.[79] High temperatures appear particularly harmful in those treated with thrombolysis,[80] although the mechanism for this remains unclear.

Although temperature appears to influence stroke prognosis,[78] stroke severity[67] and inflammation[81] may be responsible for some or all of its apparent effect. Stroke severity influences both stroke prognosis[67] and temperature[66] and the effect of temperature on outcome has been variably lost[82] or retained[83] after adjustment for stroke severity. Inflammation similarly influences both temperature[76] and stroke prognosis.[81] Again, the relationship between temperature and outcome has been variably lost[68] or retained[75] after adjustment for inflammation. It is also possible that inflammation itself is an epiphenomenon of stroke severity,[84] although this theory is not universally supported.[85] Alternatively, each of these factors could independently affect prognosis, and one study found independent associations between all three factors and early neurological worsening.[75] These relationships require clarification to enable determining which of these factors might be suitable targets for treatment. It is, of course, hoped that simply lowering body temperature poststroke might improve outcome.

To this end, antipyretic agents administered orally,[86] rectally[87] or intravenously[87] have been used to modify temperature. The effect of antipyretics varies from zero[87] to around 0.2°C.[65,88] The effect of antipyretics, however, is more marked when larger temperature rises are expected; a 0.4°C treatment effect was seen in a cohort treated within 24 h of stroke onset.[69] There is no effect from these agents at 5 days poststroke.[69] These findings suggest that antipyretic agents prevent fever rather than actively lower temperature poststroke. Clarification of the effects of these agents would facilitate the assessment of protocols that combine treatments.[89]

Active cooling techniques are highly effective at lowering body temperature and are generally employed with the aim of inducing hypothermia. The two main methods used are surface[90–92] and endovascular[93–95] cooling. Methods to selectively cool the brain,[96] for example with a cooling helmet,[97] have also been tried. Very low temperatures can be achieved with these techniques, but most protocols aim for a target temperature of 32-33°C.[94,98] This generally requires the patient to be intubated, with neuromuscular blockade used to manage shivering. Protocols avoiding the need for intubation would be more widely applicable, but the depth of hypothermia possible in awake patients is generally limited to around 35.5-36.4°C,[90,92] although a recent study achieved median core body temperatures of 33.4°C with endovascular cooling, in awake patients.[93]

Several technical issues regarding induced hypothermia require clarification before this treatment can be evaluated in large randomized trials. These issues are related to the following – the most appropriate patients to be treated, the time window for commencing temperature lowering treatments, how low the temperature should be reduced to, how long the hypothermia should be maintained and how quickly to re-warm the patient.[99]

Given the intensive resources required for monitoring patients undergoing therapeutic hypothermia and the need for large potential treatment effects to find benefit with small numbers, most studies, to date, have focused on patients with more severe stroke,[90] for example large middle cerebral artery (MCA) territory infarcts.[91,94]

There are no studies that have specifically addressed the issue of the time window for initiation of hypothermia. For practical reasons, subjects have had hypothermia initiated between 4 h[91] and 60 h[90] poststroke. It would seem likely that the maximum benefit will result from earlier initiation of treatment.

Most investigators have maintained hypothermia for one to three days,[52,90,94,95] although durations from six hours[92] to three weeks[98] have been studied. The optimal duration of hypothermia is unclear, these reports suggesting no clear benefits from either shorter or longer durations of hypothermia.

Slower rewarming seems to be safer than more rapid rewarming, there being a lower incidence of rebound elevations in Intracranial Pressure (ICP) and a lower risk of transtentorial herniation.[100]

Other side effects of these techniques include hypotension (patients often require BP support), for example, with inotropes[90,95] and pneumonia.[95]

To date, studies involving these techniques have primarily been aimed at evaluating the feasibility and safety of treatment rather than at demonstrating its effectiveness. Overall, these techniques do appear to be feasible and relatively safe.[92,95,98] Clinical endpoints have not clearly been improved with treatment, but there are studies demonstrating improvement in surrogate outcome; for example, microdialysis studies have shown lower concentrations of glycerol (a neuronal membrane component released from infarcted neurons) and lactate (indicating anerobic metabolism) with body temperatures under 34°C, and less ICP elevation[91] and cerebral edema[101] with lower body temperatures. Definitive studies are awaited.

### Intracerebral hemorrhage

Temperature rises after ICH,[67] probably more so than after an ischemic stroke.[102] The magnitude of the rise seen by Boysen and Christensen,[67] after severe (SSS < 25) ICH was about 1°C, beginning at about 4-6 h and being complete by about 12 h after the stroke. As with ischemic stroke, the temperature rise is more pronounced after severe ICH as assessed either clinically[67] or by hematoma volume.[103]

As opposed to the uncertainty surrounding temperature changes after ischemic stroke, temperature elevations after ICH seem to be mostly an epiphenomenon of the severity of the hemorrhage. Unadjusted analyses have suggested a poor prognosis, with higher temperatures.[103,104] However, analyses incorporating adjustment for stroke severity have found no association with prognosis.[67,105] One study did show that 24-48 h or >48 h with a temperature above 37.5°C was associated with a poor Glasgow Outcome Scale (GOS) score at discharge, independent of Glasgow Coma Score (GCS) and hematoma volume,[106] suggesting that there may be a dose effect.

There are a few studies examining the effects of temperature lowering after ICH. Better 3-month outcome have been seen with nasopharyngeal cooling and indomethacin, as compared with no treatment.[107] Other groups have studied surface cooling in cohorts including both ischemic stroke and ICH. These results were discussed previously.[90] Overall, however, little is known about the benefits of temperature lowering after ICH.

## Glucose

### Ischemic stroke

Glucose levels appear to rise after stroke. One study involving nondiabetic patients demonstrated a rise in median blood glucose level from 5.9 mmol/L at 2.5 h to 6.2 mmol/L at 6 h poststroke.[22] Indeed, poststroke hyperglycemia is a frequent phenomenon, with up to 50% of the patients having an initial blood glucose of above 6.0–7.0 mmol/L,[108,109] glucose levels that, in the fasting state, would be consistent with a pre-diabetic state.[110] In comparison, the prevalence of diabetes or impaired fasting glycemia is 34% in people aged over 60 years.[111] As one might expect, glucose levels poststroke are higher in patients with diabetes than in those without diabetes.[112–114]

Some have suggested that the initial poststroke hyperglycemia resolves spontaneously in the acute phase.[112,115] Others have found no such change.[108] Dysregulated glucose metabolism has also been shown to extend for months poststroke. In patients without known diabetes, half of one cohort[116] and two-thirds of another[117] had diabetes or impaired glucose tolerance at three months poststroke. At three years after stroke, two thirds of another cohort had either diabetes or a pre-diabetic state, with half of these patients unaware of their status.[118]

Hyperglycemia appears to be associated with more severe stroke, as assessed either with a clinical stroke scale[119] or by lesion volume.[120] A neuroendocrine stress response[121] and an inflammatory response[122] may also play a role in generating hyperglycemia.

Stroke location may be important, with infarction of the insular cortex, a structure involved in the autonomic control of the neuroendocrine stress response associated with hyperglycemia;[123] however this association has been refuted.[124]

Support for a neuroendocrine stress response comes from groups that have reported a correlation between glucose and cortisol levels after stroke,[17,125] although others have not found glucose to be associated with either cortisol[77] or catecholamine levels.[126]

Although not a universal finding,[127] poststroke hyperglycemia has been associated with poor outcome[128] and seems to particularly affect outcome in patients without diabetes. In the meta-analysis by Capes *et al*., the relative risk of in-hospital/30-day mortality in patients with admission hyperglycemia (>6.1–7.0 mmol/L) was 3.28 (95% CI 2.32 to 4.64) in ischemic stroke patients without diabetes, but not significantly increased in patients with diabetes.[129] Similar findings have been published since this meta-analysis.[114,130] Outcome other than mortality have also been shown to be worse in hyperglycemic patients without diabetes than in other stroke patients.[113,131]

Why hyperglycemia particularly affects stroke prognosis in patients without diabetes is unclear. Despite being apparently protective against ischemic damage *in vitro*, *in vivo* studies have consistently associated high glucose levels with harm.[132] Diabetes is associated with microcirculatory abnormalities in the brain, including arteriovenous shunting and a reduction in glucose transport across the blood-brain barrier.[133]. These processes would reduce the delivery of glucose from the blood to the brain of a patient with diabetes, thus possibly protecting them from high glucose levels after stroke.

Other factors could potentially explain the relationship between glucose and stroke prognosis. Hyperglycemia only increases growth of the infarct core in patients with surrounding hypoperfusion,[134] suggesting that hyperglycemic blood is only toxic to ischemic brain. Similarly, several studies have shown that non-ischemic brain surrounding lacunar infarcts[135] and, in turn, stroke prognosis, is unaffected[136] or perhaps even improved[137] by hyperglycemia. Conversely, Toni *et al*. found that a collateral blood supply could improve the prognosis if hyperglycemia coexists,[138] suggesting that glucose can protect ischemic brain. Several biochemical mechanisms, including excessive glutamate or lactate, vascular reactivity, or oedema formation[132] could possibly link glucose and stroke prognosis. However, these relationships remain too unclear to reconcile these apparently contradictory findings.

Hyperglycemia has a particularly potent adverse effect after thrombolysis. Hyperglycemic patients more commonly develop intracerebral hemorrhage after thrombolysis[139] and have overall poorer clinical[140] and radiological[141] outcome. Hyperglycemic patients are also less likely to recanalise with thrombolysis.[142] Even if recanalization occurs, hyperglycemic patients are more likely to deteriorate,[143] particularly if hyperglycemia occurs early after recanalization.[144]

Hyperglycemia may be merely an epiphenomenon of other underlying processes. Given the association between stroke severity and hyperglycemia,[22,119] the repeated finding that hyperglycemia has no association with prognosis after adjustment for stroke severity[82,145] suggests that in some cases hyperglycemia is an epiphenomenon of stroke severity. In other cases, however, hyperglycemia has affected prognosis independent of stroke severity.[146,147] Glucose has variably lost[148] or retained[75] an independent effect on stroke prognosis, after adjustment for IL-6 levels. Similarly, glucose has variably lost[125] or retained[149] its independent association with stroke prognosis after adjustment for cortisol levels. As glucose is associated with both inflammation[75,122] and cortisol levels,[17,125] and as stroke prognosis is also associated with both inflammation[81] and cortisol,[77] it is possible that these factors drive stroke prognosis and that hyperglycemia is merely an epiphenomenon of one or both of these factors.

Given the frequency of hyperglycemia and its effect on outcome, glucose-lowering therapy has potential as a widely applicable treatment after stroke. Insulin, specifically glucose-potassium-insulin (GKI) infusions, have been shown to be feasible and safe in acute stroke patients.[150,151] Insulin appears to have beneficial effects, including anti-inflammatory, antioxidant and nitric oxide effects, which are independent of its ability to lower glucose levels, and which may be beneficial in stroke.[152]

Unfortunately, the large Glucose Insulin in Stroke Trial (GIST-UK)[51] was terminated prematurely due to slow recruitment, after 933 patients were randomized to either GKI or intravenous saline. Being correspondingly underpowered, the study failed to identify a treatment effect on mortality or other outcome. Further studies are clearly warranted.

There have been few studies with other agents used in the treatment of diabetes, although sulfonylureas were not shown to affect stroke prognosis in one trial.[153]

### Intracerebral hemorrhage

Hyperglycemia after ICH is less well-characterized than after ischemic stroke. Apart from diabetes,[154] the most significant determinant of hyperglycemia is the severity of the ICH, as assessed by the hematoma size[155] or other markers of severity.[154]

In the meta-analysis by Capes *et al*., admission hyperglycemia was not associated with higher mortality in unadjusted analyses of either diabetic or nondiabetic ICH patients.[129] Since this meta-analysis, one group found that hyperglycemia had no association with outcome after adjustment for ICH volume and growth, two strong predictors of outcome after ICH.[62] Other studies, however, have shown an independent effect from glucose, even after adjusting for the volume[106,156] or other markers of mass effect[154] of the ICH. These subsequent results suggest that glucose does have an independent effect on ICH prognosis.

There are no treatment trials specifically addressing the effects of glucose lowering in ICH patients.

## Oxygenation

Hypoxia is frequently reported after stroke, although the frequency depends on the definition used. Pulse oximetry identified arterial oxygen saturations (SaO2) <90% for >10% of the recording time in 20% of one stroke cohort,[157] while 63% of another cohort had SaO2 <96% for at least 5 min.[158]

Hypoxia in stroke patients is commonly associated with co-morbidities such as respiratory tract infections and cardiac failure.[157] It also occurs overnight, with SaO2 falling below 90% for >30 min in 25% of patients in one study.[159] Hypoxia does not seem to be related to stroke severity.[157]

Transient hypoxia has been observed during routine maneuvers such as MRI scanning (18% patients had SaO2 <90% for at least 1 min);[160] bed transfers (SaO2 fell ≥ 3% in 18% patients)[157] and nasogastric tube insertion, when difficult or prolonged (SaO2 fell below 90% in 21% patients).[161] Hypoxia is not related to overnight nasogastric tube feeding.[162] Hypoxia may[163] or may not[164] occur with oral feeding; this inconsistency may be due to the use of differing criteria for classifying patients as safe to feed orally.

Posture may affect oxygenation, with higher SaO2 levels when sitting upright or semi-recumbent, as compared with fully recumbent posture.[165] Positioning may be more likely to affect oxygenation in stroke patients who have coexisting respiratory problems.[166]

It is generally assumed that hypoxia carries a poor prognosis and is rarely left untreated in clinical practice or research settings. In a mixed cohort of ischemic and hemorrhagic stroke, there was a univariate association between hypoxia (SaO2 < 90% for > 10% of the recording time) and death at three months poststroke, but this was nonsignificant after adjusting for stroke severity.[157] There is a corresponding lack of evidence that correction of hypoxia improves stroke prognosis.

Oxygen supplementation has been studied as a way to improve stroke outcome in normoxic patients. Understandably, most research has focused on ischemic stroke, where oxygen supplementation is seen as having a potential for neuroprotection. Potential benefits suggested by the results of animal studies include suppression of excitotoxicity, oxidative and nitrosative stress, inflammation, and apoptosis.[167] Despite theoretical concerns about the production of harmful superoxide free radicals and hyperoxia-induced vasoconstriction, some human studies suggest an overall beneficial effect. Indeed, oxygen-induced vasoconstriction in nonischemic areas has been shown to shunt blood towards ischemic areas, thus paradoxically improving perfusion.[168]

Oxygen supplementation using both hyperbaric (HBO) and normobaric oxygen (NBO) to improve outcome in normoxic patients has been studied. Hyperbaric oxygen (HBO) has been more widely studied and the early studies showed occasional dramatic improvements temporally related to HBO therapy, with some of these improvements occurring even several weeks and months after stroke.[169,170] More recent studies have focused on acute stroke, but the results have either been neutral[171] or have suggested better outcome in the non-HBO group.[172]

Various methodological explanations for these conflicting results have been offered. The optimum treatment pressure remains unclear, with pressures from 1.5 atmospheres absolute (ATA) to 2.5 ATA promoted as being the most appropriate.[167,173] The use of oxygen therapies by control groups, usually in an attempt to maintain blinding, may have offered some treatment effect and, therefore, may also have affected the results in some studies.[171,172] The initiation of HBO therapy beyond the first few hours after stroke has also been criticized, because extrapolation from other stroke therapies[173] and animal studies[174] suggests that poor outcome from treatment this late after stroke are to be expected.

Normobaric oxygen (NBO) is capable of increasing the SaO2 in normoxic stroke patients,[175] but unselected patients appear to do poorly with NBO therapy.[176] Patients presenting with large MCA territory infarcts appeared to benefit from oxygen supplementation (FiO2 40% by Venturi mask), when treated within 48 h of stroke, perhaps because these patients are more likely to have a penumbra.[177] Similarly, diffusion weighted imaging (DWI) volumes were smaller during treatment with high-flow (45 L/min) mask oxygen given within 12 h of stroke in patients with MRI evidence of perfusion-diffusion mismatch.[168] As with HBO, the dose and timing issues are unclear for NBO therapy also.

Oxygen therapy might protect the ischemic penumbra by suppressing harmful processes such as edema,[178] inflammation and apoptosis.[167] While NBO does not appear to provide permanent protection from recruitment of the ischemic penumbra into the infarct core,[179] it might be used to maintain the penumbra whilst awaiting reperfusion and thus prolonging the therapeutic time window for reperfusion therapies.[168] Applying HBO in the same way poses significant logistical challenges,. Treatment would be restricted to a small subgroup of the already small percentage of thrombolysis-eligible patients.[180]

## Monitoring of physiology in the stroke unit

While stroke-unit care reduces mortality and improves functional independence,[1] the mechanisms for this benefit remain unclear. One mode that stroke-unit care may help patients is through careful monitoring and optimization of physiological variables.[181]

Stroke units might facilitate the maintenance of physiological homeostasis by bringing together medical, nursing and other staff experienced in stroke care; the necessary equipment for monitoring physiological parameters; and protocols to manage aberrant physiology. Monitoring in stroke units also facilitates data collection for subsequent refinement of management protocols.

There is some evidence that intensive physiological monitoring in a stroke unit improves outcome.[2,3] In a randomized study of 54 ischemic stroke patients,[2] continuous monitoring of BP, electrocardiogram (ECG), body temperature, and pulse oximetry for at least 48 h improved 3-month mortality, as compared with 6th-hourly manual measurements of BP, temperature, and heart rate and intermittent pulse oximetry (3.7% *vs* 25.9%; *P*=0.05). The same protocol for managing abnormal physiology was used in both groups, suggesting that early detection and management of abnormalities through continuous monitoring improved outcome. Improved outcome was also observed in a nonrandomized comparison between continuous monitoring of BP, ECG, temperature, oxygen saturation, respiratory frequency and electroencephalogram (EEG), and intermittent monitoring of BP, heart rate, and temperature. Aberrant physiology was more frequently detected in the continuously monitored group (64% *vs* 19%; *P*<0.0001), perhaps leading to more timely intervention and resulting in improved outcome.[3] One randomized study of 206 ischemic stroke patients showed that less DBP variability was associated with more frequent discharge to home at six weeks, although it is unclear whether this was due to more frequent monitoring or earlier mobilization in the stroke unit.[182]

As can be seen from the above discussion, the optimum strategies for managing these physiological variables remain uncertain. Many stroke units have developed local management protocols guided by the available evidence, informed by experience, and modified according to the resources available locally. Internationally, published evidence-based stroke-management guidelines provide some guidance.

## What do published guidelines say about management of physiological variables?

Table 1 summarizes the recommendations from published American, European, and Australian stroke treatment guidelines, regarding management of physiological parameters after acute stroke. There is general consistency across the guidelines, with some minor differences, generally in areas in which there is a lack of strong evidence to guide practice.

The guidelines generally recommend tolerating higher than usual BP after both ischemic stroke and intracerebral hemorrhage. Excessive elevations should be treated, but thresholds for initiating treatment and targets for treatment vary. All caution should be taken against aggressive and abrupt BP reductions. Treatment of fever with antipyretic agents is recommended, with a search for infection and antibiotics when appropriate. Treatment of hyperglycemia for both forms of stroke is recommended, although thresholds for initiating therapy vary. Treatment of hypoglycemia is advised. Hypoxic stroke patients should receive oxygen therapy, but evidence is insufficient for the guidelines to recommend oxygen therapy for normoxic patients.

**Table 1:** Summary of recommendations from published guidelines regarding management of physiological variables after acute stroke

Physiological Variable	USA[183,184]	Europe[185,186]	Australian[187]
Blood Pressure	IS: Patients with markedly elevated BP (SBP > 220 mm Hg or DBP > 120 mm Hg) may have their BP lowered. A reasonable goal would be to lower BP by ~15%.	IS: Routine BP lowering is not recommended, except for extremely elevated values (SBP > 200-220 mm Hg or DBP > 120 mm Hg).	IS: If extremely high BP (> 220/120 mm Hg), institute or increase antihypertensive therapy, but BP should be cautiously reduced (by no more than 10-20%)
	Patients for tPA should be stabilized with SBP < 185 mm Hg and DBP < 110 mm Hg before starting treatment. Hypotension should be corrected (eg, hypovolemia with fluid replacement).	Recommended target BPs: Prior hypertension: 180/100-105 mm Hg No prior hypertension: 160- 180/90- 100 mm Hg Thrombolysis: SBP < 180 mm Hg ICH: Treatment is recommended if BP is above the following levels: (i) Patients with hypertension: SBP > 180 mm Hg and/or DBP > 105 mm Hg. (Target BP 170/100 mm Hg or MAP 125 mm Hg). (ii) Patients without hypertension: SBP > 160 mm Hg and/or DBP > 95 mm Hg. (Target BP 150/90 mm Hg or a MAP 110 mm Hg). (iii) Avoid reducing MAP by > 20%. (iv) In patients undergoing monitoring for increased ICP ensure CPP > 70 mm Hg.	ICH: In patients with a history of hypertension, MAP should be maintained < 130 mm Hg
	ICH: (i) If SBP > 200 mm Hg or MAP > 150 mm Hg, consider aggressive BP reduction. (ii) If SBP > 180 mm Hg or MAP > 130 mm Hg and evidence/suspicion of elevated ICP, consider monitoring ICP and reducing BP to keep CPP >60-80 mm Hg. (iii) If SBP > 180 mm Hg or MAP > 130 mm Hg and no evidence/suspicion of elevated ICP, consider modest BP reduction (target BP 160/90 mm Hg or MAP 110 mm Hg).		
Temperature	IS and ICH: Sources of fever should be treated and antipyretic medications should be administered to lower temperature in febrile patients	IS and ICH: Treatment of body temperature ≥ 37.5°C is recommended. Search for a possible infection and consider appropriate antibiotic therapy.	IS and ICH: Antipyretic therapy, comprising regular paracetamol and/or physical cooling measures, should be used routinely where fever occurs
Glucose	IS and ICH: Persistent hyperglycemia with glucose > 185 mg/dl (10.2 mmol/l), and possibly > 140 mg/dl(7.8 mmol/l), should probably trigger administration of insulin. Hypoglycemia should be treated to achieve normoglycemia.	IS and ICH: Treatment of blood glucose > 200 mg/dl (11 mmol/l) with insulin titration is recommended. Immediate correction of hypoglycemia is recommended.	IS and ICH: Patients with hyperglycemia should have their blood glucose level monitored and appropriate glycemic therapy instituted to ensure euglycemia, especially if the patient is diabetic. Intensive early maintenance of euglycemia is currently not recommended.
			Hypoglycemia should be avoided.
Oxygenation	IS: Hypoxic patients should receive supplemental oxygen (maintain SaO_2_ ≥ 92%) Nonhypoxic patients do not need supplemental oxygen therapy ICH: No specific advice other than to ensure adequate oxygenation	IS and ICH: Oxygen supplementation is recommended if SaO_2_ < 92%	IS and ICH: Patients who are hypoxic should be given oxygen supplementation (no SaO_2_ threshold specified).

IS: Ischemic Stroke, ICH: intracerebral hemorrhage, BP: blood Pressure, SBP: systolic blood pressure, DBP: diastolic blood pressure, MAP: mean arterial pressure, CPP: cerebral perfusion pressure, ICP: intracranial pressure, SaO_2_: arterial oxygen saturation.

(Please refer to full text of guidelines for details of specific recommendations)

## Conclusions

Our understanding of the changes in BP, temperature, glucose, and oxygen levels after stroke remains incomplete. Further study should clarify the natural history of acute physiological changes, the mechanisms behind these changes, and the effect these changes have on outcome. If physiological abnormalities contribute significantly to poststroke outcome, they offer an obvious target for treatment. Intensive early monitoring and control of physiological parameters might then become the standard of care in the stroke units of the 21st century.
